# Targeted next-generation sequencing reveals high frequency of mutations in epigenetic regulators across treatment-naïve patient melanomas

**DOI:** 10.1186/s13148-015-0091-3

**Published:** 2015-06-09

**Authors:** Jonathan J. Lee, Lynette M. Sholl, Neal I. Lindeman, Scott R. Granter, Alvaro C. Laga, Priyanka Shivdasani, Gary Chin, Jason J. Luke, Patrick A. Ott, F. Stephen Hodi, Martin C. Mihm, Jennifer Y. Lin, Andrew E. Werchniak, Harley A. Haynes, Nancy Bailey, Robert Liu, George F. Murphy, Christine G. Lian

**Affiliations:** Department of Pathology, Brigham and Women’s Hospital, Harvard Medical School, 221 Longwood Avenue, EBRC Suite 401, Boston, MA 02115 USA; Melanoma Center, Dana–Farber Cancer Institute, Harvard Medical School, 450 Brookline Ave., Boston, MA 02215-5450 USA

**Keywords:** Melanoma, Next-generation sequencing (NGS), Epigenetics, *MECOM* (MDS1 and EV1 complex locus), *MLL2*, Ten-eleven translocation (TET), Isocitrate dehydrogenase 2 (IDH2), 5-hydroxymethylcytosine, DNA demethylation

## Abstract

**Background:**

Recent developments in genomic sequencing have advanced our understanding of the mutations underlying human malignancy. Melanoma is a prototype of an aggressive, genetically heterogeneous cancer notorious for its biologic plasticity and predilection towards developing resistance to targeted therapies. Evidence is rapidly accumulating that dysregulated epigenetic mechanisms (DNA methylation/demethylation, histone modification, non-coding RNAs) may play a central role in the pathogenesis of melanoma. Therefore, we sought to characterize the frequency and nature of mutations in epigenetic regulators in clinical, treatment-naïve, patient melanoma specimens obtained from one academic institution.

**Results:**

Targeted next-generation sequencing for 275 known and investigative cancer genes (of which 41 genes, or 14.9 %, encoded an epigenetic regulator) of 38 treatment-naïve patient melanoma samples revealed that 22.3 % (165 of 740) of all non-silent mutations affected an epigenetic regulator. The most frequently mutated genes were *BRAF*, *MECOM*, *NRAS*, *TP53*, *MLL2*, and *CDKN2A*. Of the 40 most commonly mutated genes, 12 (30.0 %) encoded epigenetic regulators, including genes encoding enzymes involved in histone modification (*MECOM*, *MLL2*, *SETD2*), chromatin remodeling (*ARID1B*, *ARID2*), and DNA methylation and demethylation (*TET2*, *IDH1*). Among the 38 patient melanoma samples, 35 (92.1 %) harbored at least one mutation in an epigenetic regulator. The genes with the highest number of total UVB-signature mutations encoded epigenetic regulators, including *MLL2* (100 %, 16 of 16) and *MECOM* (82.6 %, 19 of 23). Moreover, on average, epigenetic genes harbored a significantly greater number of UVB-signature mutations per gene than non-epigenetic genes (3.7 versus 2.4, respectively; *p* = 0.01). Bioinformatics analysis of The Cancer Genome Atlas (TCGA) melanoma mutation dataset also revealed a frequency of mutations in the 41 epigenetic genes comparable to that found within our cohort of patient melanoma samples.

**Conclusions:**

Our study identified a high prevalence of somatic mutations in genes encoding epigenetic regulators, including those involved in DNA demethylation, histone modification, chromatin remodeling, and microRNA processing. Moreover, UVB-signature mutations were found more commonly among epigenetic genes than in non-epigenetic genes. Taken together, these findings further implicate epigenetic mechanisms, particularly those involving the chromatin-remodeling enzyme *MECOM*/EVI1 and histone-modifying enzyme *MLL2*, in the pathobiology of melanoma.

**Electronic supplementary material:**

The online version of this article (doi:10.1186/s13148-015-0091-3) contains supplementary material, which is available to authorized users.

## Background

Despite advancements in our understanding of the molecular mechanisms underlying melanogenesis, disease progression, and therapeutic response, melanoma remains one of the deadliest forms of human malignancy [1]. Identification of frequent mutations in the gene encoding the serine/threonine-protein kinase B-Raf (*BRAF*) in melanoma [2] led to the development of targeted inhibitors with improved survival demonstrated in phase III clinical trials demonstrating improved overall survival [3]. However, soon after the discovery of frequent *BRAF* V600E mutations in melanoma, the same mutation was found at higher frequencies in benign and dysplastic melanocytic nevi [4]. Since then, *BRAF* mutations, alone, have become understood to be “insufficient” to induce tumor progression beyond the benign melanocytic nevus stage [5].

Despite initial clinical response after targeted, single agent therapy, the subsequent development of resistance in patients being treated for metastatic melanoma is, essentially, universal [6]. This is largely attributed to the development and progression of chemoresistant subpopulations, enabled, in part, by unique biological characteristics inherent to malignant melanoma [7]. Complicating matters is that approximately one in every five patients harboring *BRAF* V600 mutant melanoma will have disease that is intrinsically resistant to *BRAF* inhibition and will be found to have progressed on therapy at first follow-up assessment [6]. While the combined use of targeted therapies (BRAF and/or MEK inhibition) [7–9] as well as immunotherapies (monoclonal antibodies directed at CTLA-4 and PD-1/PD-L1) have shown significantly improved outcomes for melanoma patients with advanced disease [10, 11], evidence implicating dysregulated epigenetic mechanisms in the pathogenesis of melanoma and other malignancies is also accumulating at a rapid pace [12, 13]. This growing body of literature has significant translational potential to elucidate novel pathogenic mechanisms in melanoma and deserves thorough investigation, as therapeutically targeting epigenetic mechanisms [14, 15] in combination with other targeted or immunotherapeutic modalities may be necessary to achieve sustainable clinical remission [16–18].

Recent advancements in next-generation sequencing (NGS) technologies have facilitated whole-genome, whole-exome, and whole-transcriptome analyses that could enable personalized diagnostic and therapeutic strategies. These technologies have demonstrated substantial power and sensitivity in identifying novel mutated genes in melanoma and have even been used to gain insight into mechanisms of primary (intrinsic) [17] and secondary (acquired) chemoresistance in select melanoma patients [7, 19]. Moreover, developments in computational processing and statistical analytics have also enabled the dissection of “driver” mutations (those that confer a fitness advantage to a particular tumor cell) from “passenger” mutations (those that do not) [20]. Indeed, such efforts to enhance molecular precision while characterizing the genetic and epigenetic landscape within an individual patient’s melanoma may be necessary to help guide combinatorial therapies [21].

Herein, we describe the targeted NGS platform devised at our institution to sequence 275 known and investigative cancer genes (“Oncopanel”, Brigham and Women’s Hospital and Dana–Farber Cancer Institute) in patients being evaluated for the management of melanoma and report the frequency and nature of gene mutations identified. A list of the genes tested for by the Oncopanel platform is provided in Additional file 1. A substantial fraction (14.9 %, 41 of 275) of these genes encode either well-established or recently-described epigenetic regulators, including those involved in DNA methylation and demethylation, histone modification, chromatin remodeling, and non-coding RNAs. In light of rapidly accumulating evidence for the involvement of dysregulated epigenetic mechanisms in melanoma pathogenesis [12, 13], we sought, specifically, to characterize the prevalence and nature of mutations in this select panel of epigenetic regulators within our cohort of patient melanoma samples.

## Results

### Patient demographic, clinical, and histopathologic information

A total of 38 patient melanoma samples (*n* = 38), each obtained from 38 unique patients, were available for analysis. Of these, 13 were primary cutaneous melanomas (*n*_P_ = 13) and the remaining 25 were obtained from metastatic sites (*n*_M_ = 25). Patients ranged in age from 21 to 83 years (mean, 60.9; median, 62; SD, 13.06). Primary cutaneous melanoma samples ranged in Breslow depth and mitotic rate from 0.67 to 7.50 mm (mean, 3.66 mm; median, 3.1; SD, 1.94) and from 1 to 17 mitoses/mm^2^ (mean, 5.3; median, 4; SD, 4.5). Six of the primary melanomas (46.2 %, 6 of 13) had histologic evidence of ulceration. Of the patients whose primary melanomas had been sequenced, five (38.5 %) had also gone on to develop metastatic disease. The vast majority of metastatic melanoma tissues samples were obtained from lymph nodes (44 %, 11 of 25), while the remaining metastases were obtained from the thorax (i.e., lung, chest wall, 16 %, 4 of 25), abdomen (i.e., mesentery, adrenal gland, 4 of 25, 16 %), central nervous system (i.e., brain, brainstem, 12 %, 3 of 25), or subcutaneous tissue (i.e., in transit metastasis) (12 %, 3 of 25).

### General mutation distribution and characteristics

**Fig. 1:**
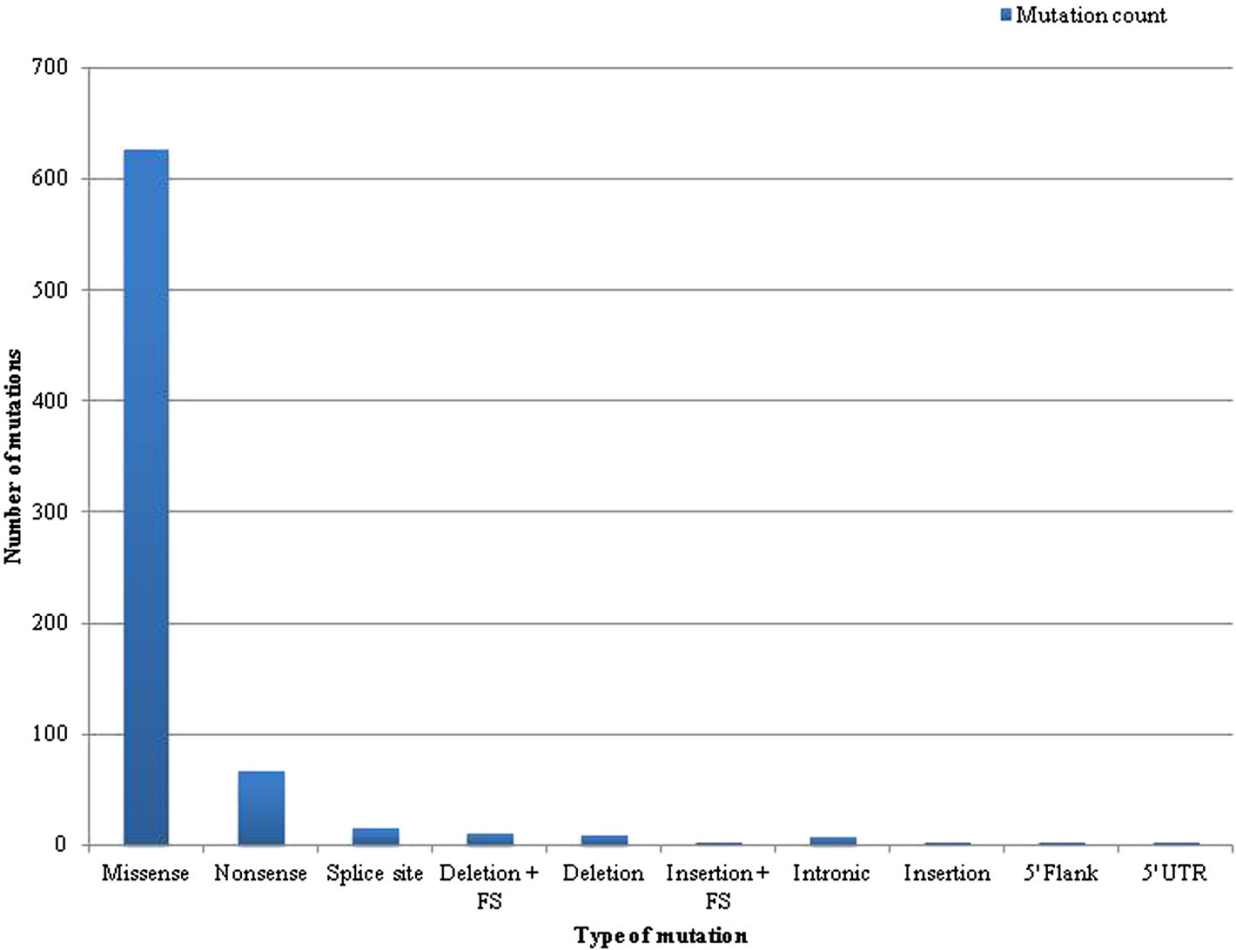
Bar graph summarizing distribution of mutation types in our 38 patient melanoma samples

Collectively across all 38 patient samples, a total of 740 non-silent mutations were identified in 204 of the 275 (74.2 %) genes originally tested for. An average of approximately 20 mutations (median, 15.5; range, 3 to 132; standard deviation, 21.5) was identified per patient melanoma sample. A graph summarizing the distribution of mutation types is shown in Fig. 1. The vast majority of mutations were missense mutations (84.7 %, 627 of 740), followed by nonsense mutations (8.9 %, 66 of 740), insertions or deletions resulting in frameshift (2.0 %, 14 of 740), and splice site mutations (2.0 %, 15 of 740). The largest percentage of nonsense mutations occurred in well-known tumor suppressor genes *NF1* (12.1 %, 8 of 66), *CDKN2A* (10.6 %, 7 of 66), and *TP53* (9.1 %, 6 of 66).

**Fig. 2:**
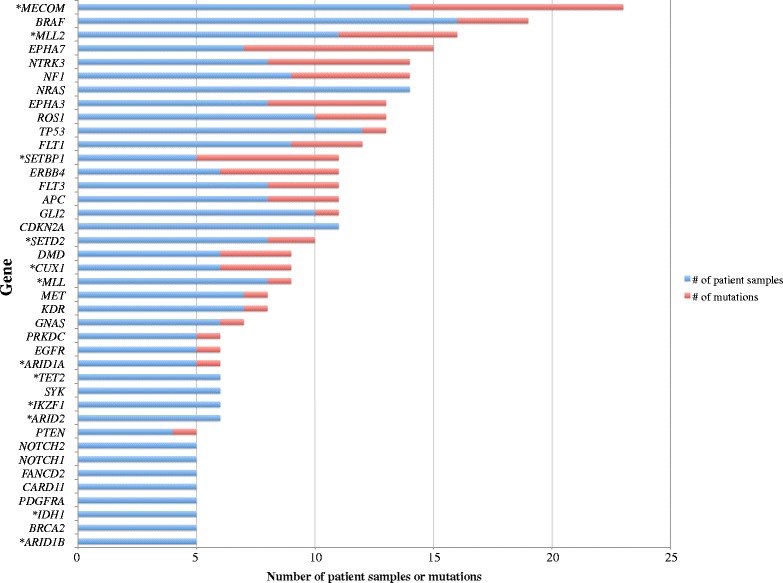
Chart summarizing the top 40 most frequently mutated genes. *Epigenetic gene

The top 40 most frequently mutated genes, as determined by the number and frequency of total mutations, are graphically represented in Fig. 2. *BRAF* (42.1 % of patient samples, 16 of 38), *MECOM* (36.8 %, 14 of 38), *NRAS* (36.8 %, 14 of 38), *TP53* (31.6 %, 12 of 38), *MLL2* (29.0 %, 11 of 38), as well as *CDKN2A* (29.0 %, 11 of 38) were among the most commonly mutated genes among the patient melanoma samples. Of these genes, *MECOM*, *BRAF*, and *MLL2* harbored the greatest number of total mutations (23, 19, 16, respectively).

### High frequency of mutations identified in key epigenetic regulators

**Fig. 3:**
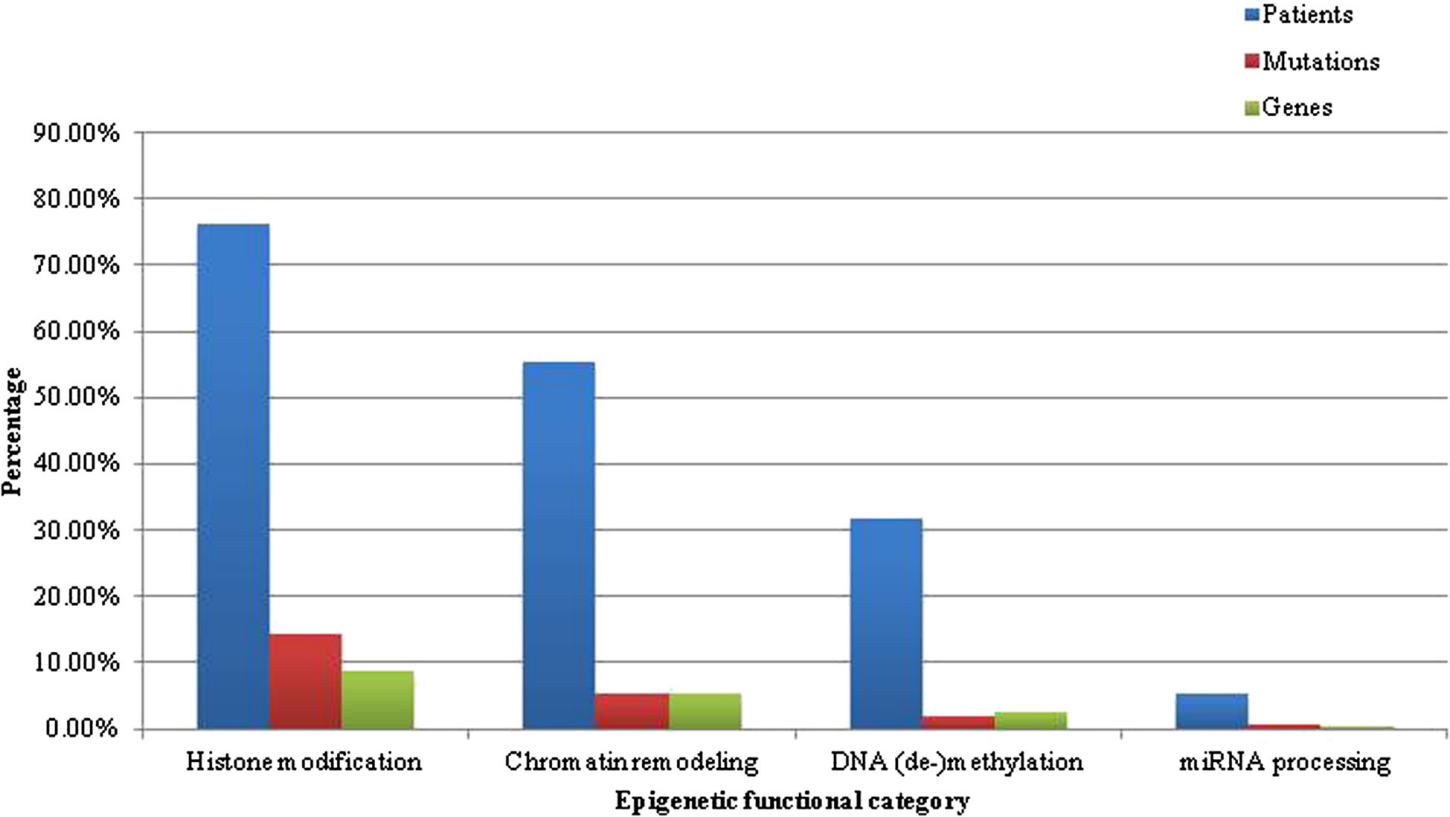
Frequency of mutated epigenetic genes organized by functional epigenetic category

Of interest, 22.3 % (165 of 740) of all mutations occurred in genes encoding epigenetic regulators. Mutations in genes encoding histone-modifying proteins were the most common (64.2 %, or 106 of 165 epigenetic gene mutations, which accounted for 14.3 %, or 106 of all 740 identified mutations), including *MECOM* and *MLL2* listed above, followed by chromatin remodeling proteins (24.2 %, 40 of 165), DNA methylation/demethylation enzymes (9.1 %, 15 of 165), and enzymes involved in miRNA processing (2.4 %, 4 of 165). A summary of the frequency of mutated epigenetic genes categorized by their functional epigenetic classification is illustrated in Fig. 3. At least one mutation in an epigenetic gene was found in 92.1 % (35 of 38) of patients, and 25 of these patients (65.7 % of all samples) had more than one epigenetic regulatory gene mutated.

Of all the mutated genes identified, 17.2 % (35 of 204) encoded epigenetic regulators, whereas 14.9 % (41 of 275) of the genes tested by our Oncopanel were epigenetic in nature. Moreover, within the top 40 most frequently mutated genes, 30.0 % (12 of 40) of the genes encoded epigenetic regulators (Fig. 2). A two-sample *z* test comparing the proportion of epigenetic regulators within the top 40 (30 %, 12 of 40) compared to that within the original panel of tested genes (14.9 %, 41 of 275) revealed a statistically significant difference (*p* = 0.017, *z* score = 2.4) between these two groups. Ten of 12 (81.2 %) of the epigenetic genes within the top 40 were found to encode either histone-modifying proteins (e.g., *MECOM*, *MLL2*, *SETD2*) or subunits of chromatin-remodeling complexes (e.g., *ARID1B*, *ARID2*). Two of 12 (16.7 %) of these epigenetic genes within the top 40 encode enzymes involved in active DNA demethylation (*TET2*, *IDH1*). Furthermore, 30.8 % (4 of 13) of all genes containing an insertion or deletion resulting in frameshift encoded epigenetic regulators, including histone-modifying enzymes (*SETD2*, *CREBBP*, *MLL*) and DNA methyltransferase 3A *DNMT3A*.

### Analysis of epigenetic mutations and key clinicopathologic parameters

**Fig. 4:**
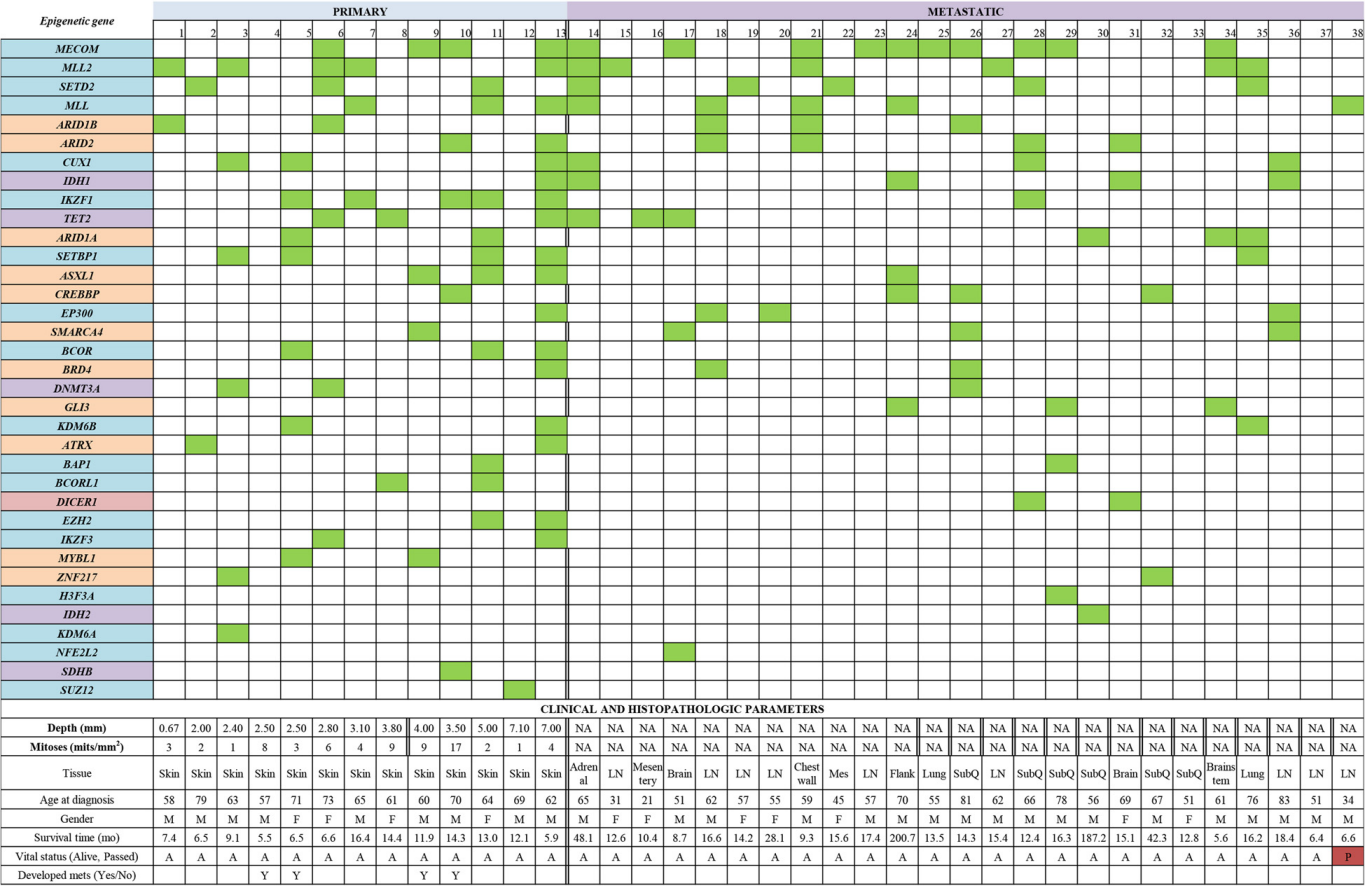
Presence of mutations in epigenetic regulators, organized by patient melanoma sample and key primary cutaneous melanoma histopathologic parameters

**Fig. 5:**
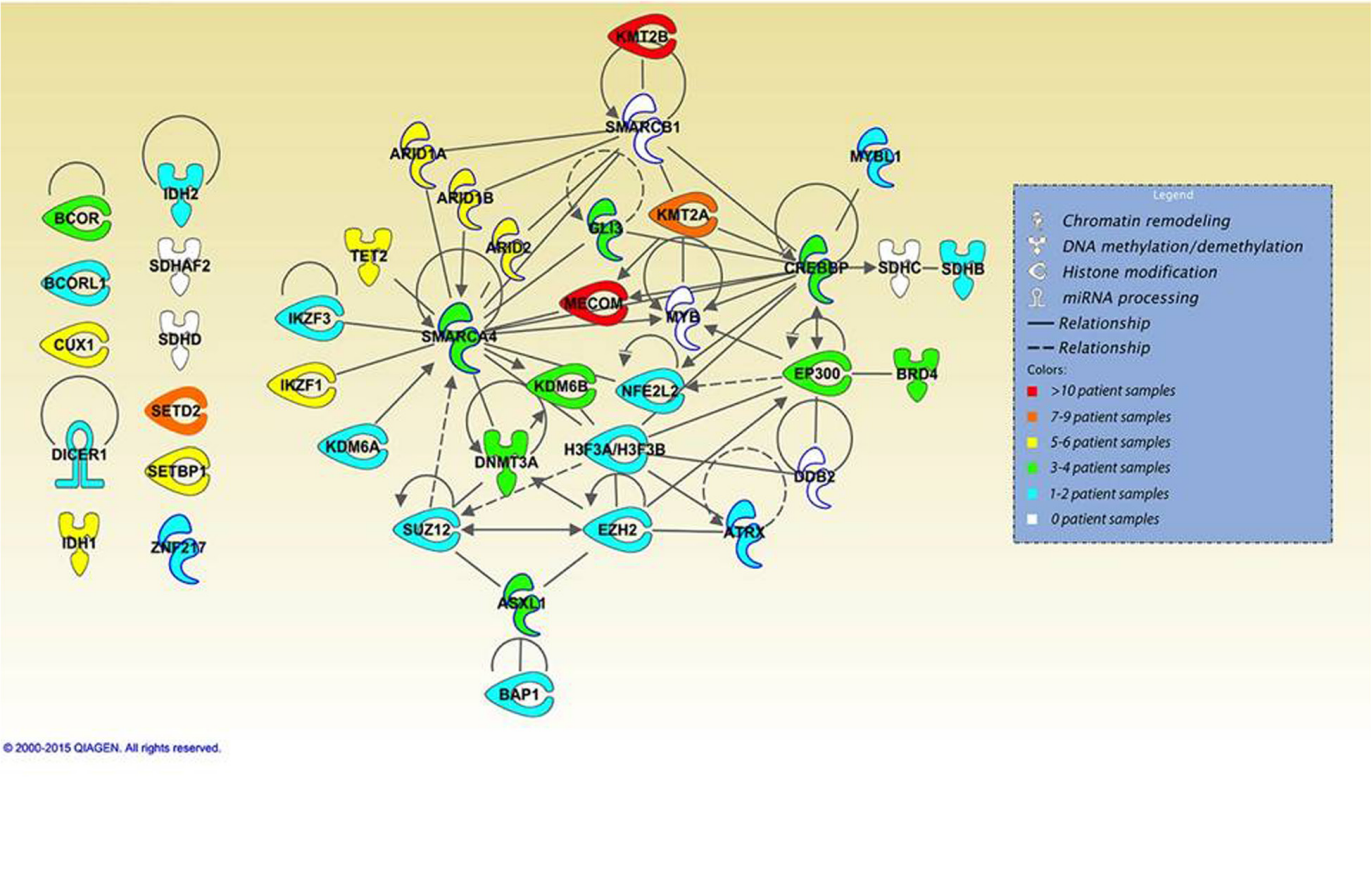
Ingenuity Pathway Analysis® of the 41 epigenetic regulators sequenced by our targeted next-generation sequencing platform (Oncopanel, BWH/DFCI). Unique shape indicates key epigenetic function while the color reflects the prevalence of mutations in that gene

A summary graphic illustrating the spectrum of epigenetic genes mutated within each patient melanoma sample as well as the clinical and histopathologic data corresponding to each patient’s case is presented in Fig. 4. Our analysis did not identify statistically significant relationships between mutations in specific epigenetic genes and key histologic parameters of primary cutaneous melanoma, such as the Breslow depth, mitotic rate, or the presence/absence of ulceration; nor did it reveal any specific epigenetic mutational signature of either primary or metastatic melanomas within the limited number of samples in our study. Nonetheless, ingenuity pathway analysis revealed a complex mechanistic interplay between several of our most frequently mutated epigenetic regulators and the prevalence of mutations in each epigenetic regulator is also graphically represented (Fig. 5).

### High frequency of UVB-pattern mutations particularly found among key epigenetic regulators

**Fig. 6:**
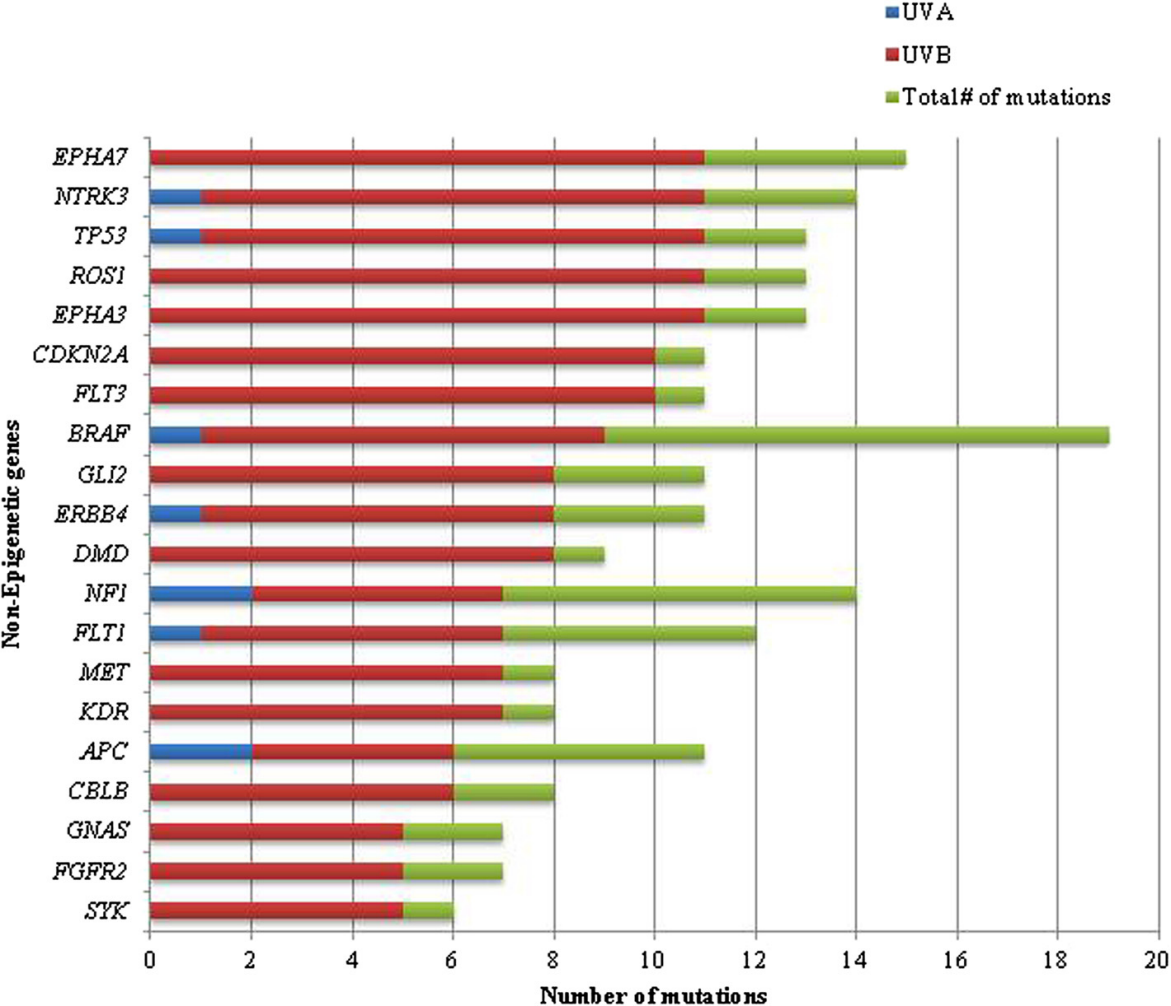
Spectrum of UV-signature mutations among non-epigenetic genes. Note the low frequency of UVB-signature mutations in *BRAF*

**Fig. 7:**
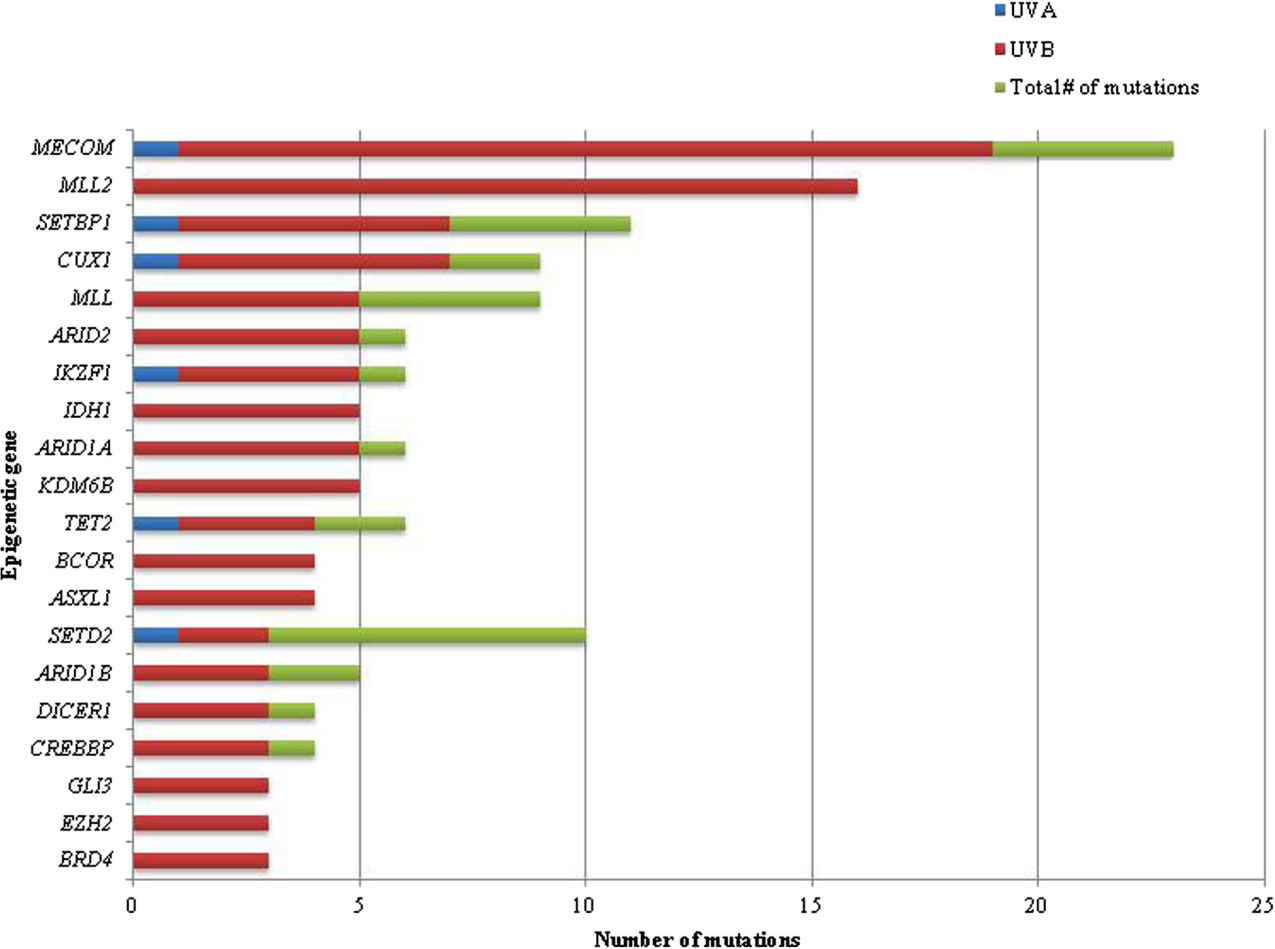
Spectrum of UV-signature mutations among epigenetic genes. Note the high frequency (100 %) of UVB-signature mutations among the gene encoding histone lysine methyltransferase, *MLL2* (*KMT2D*)

Of all the mutations identified, 73.1 % (541 of 740) bore the signature of UVB damage. A single C > T nucleotide substitution was the most common (70.3 %, 520 of 740), followed by CC > TT tandem dinucleotide mutation (2.2 %, 12 of 541), and C > T missense mutations within a dinucleotide substitution (1.7 %, 9 of 541). In contrast, only 4.9 % (36 of 740) of all mutations were G > T single nucleotide variant transitions, the signature of UVA-induced DNA damage. The genes with the greatest number of total UVB-signature mutations encoded epigenetic regulators, including *MLL2* (100 %, 16 of 16) and *MECOM* (82.6 %, 19 of 23). The spectrum of UV-signature mutations in non-epigenetic and epigenetic genes is provided in Figs. 6 and 7, respectively.

**Fig. 8:**
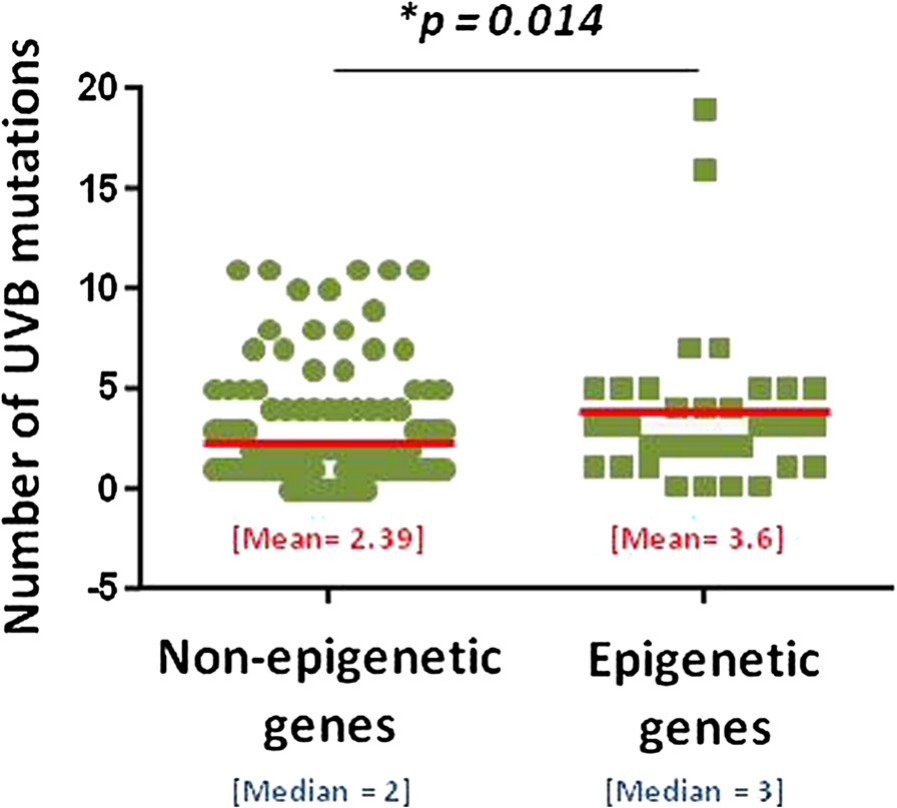
Distribution of UVB-signature mutations among non-epigenetic genes and epigenetic genes. Collectively, epigenetic genes harbored a significantly greater number of mean UVB-signature mutations than non-epigenetic genes. Turquoise bar reflects the median among both distributions

Collectively, non-epigenetic genes harbored a mean of 2.4 UVB-signature mutations per gene (median, 2; mode, 1; standard deviation, 2.5) whereas epigenetic genes harbored an average of 3.7 (median, 3; mode, 3; standard deviation, 3.9) (Fig. 8). An unpaired, two-sample *t* test comparing the average number of UVB-signature mutations per gene in non-epigenetic versus epigenetic genes revealed a statistically significant difference (*p* = 0.014) between the two groups. Of note, 81.8 % (9 of 11) of *CDKN2A* mutations and 84.6 % (11 of 13) of *TP53* mutations bore the UVB-signature. Interestingly, none of the *NRAS* mutations (0 of 14) and only 47.4 % (9 of 19) of *BRAF* mutations resembled the UVB-signature mutation.

### Bioinformatics analysis of existing melanoma mutation datasets

**Fig. 9:**
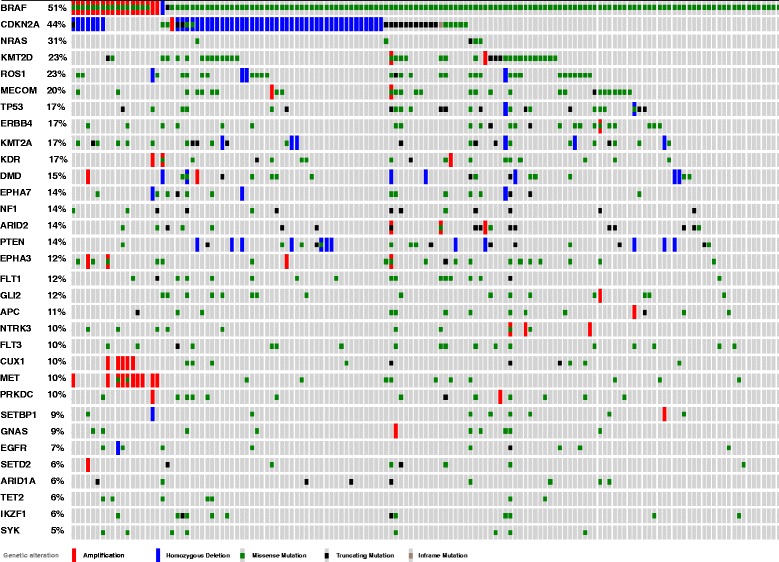
Distribution of mutations in our “Top 40” genes among TCGA cutaneous melanomas (*n* = 278) (data publicly accessible via http://www.cbioportal.org)

**Fig. 10:**
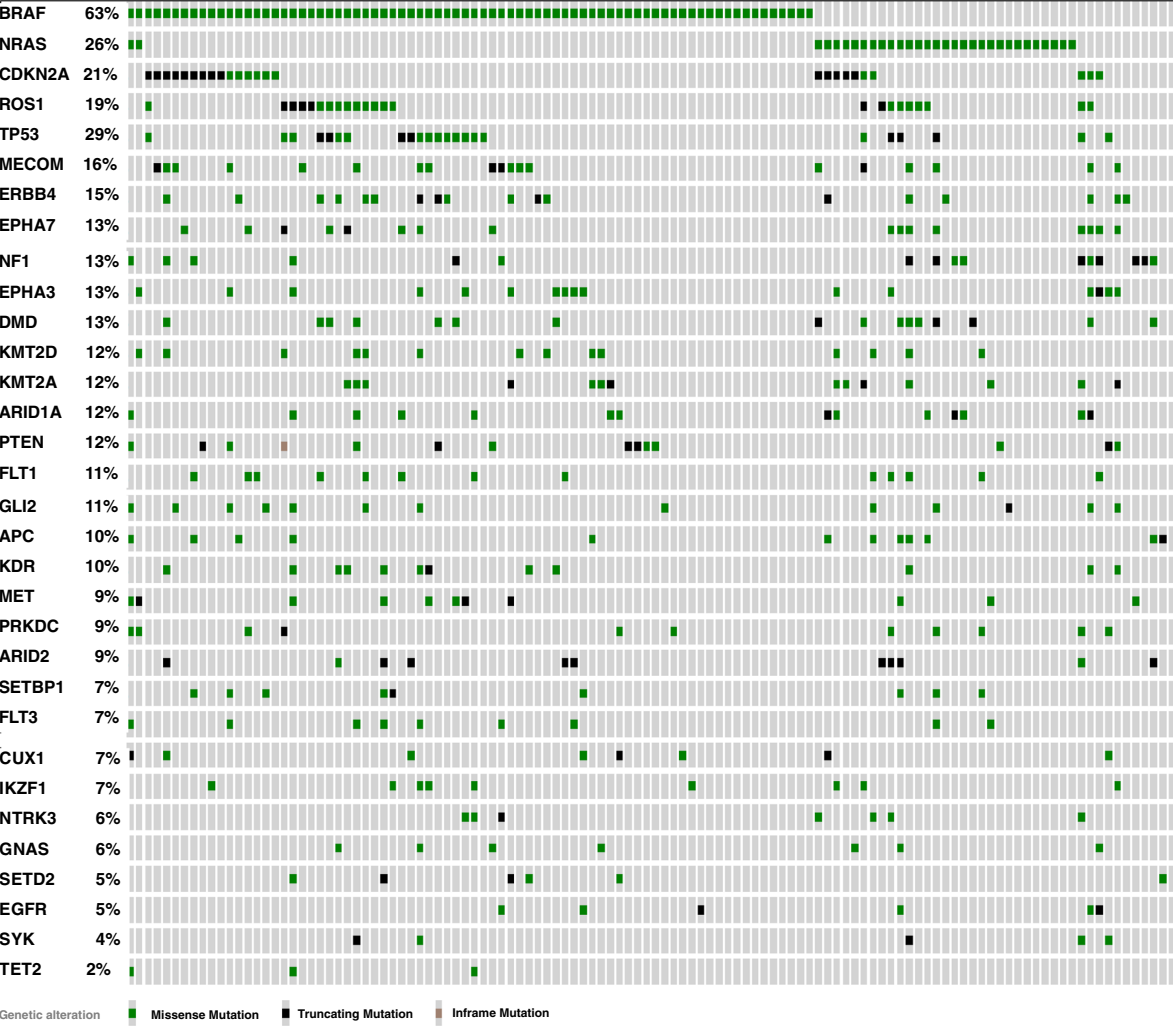
Distribution of mutations in our “Top 40” genes among the Broad Institute cutaneous melanoma samples (*n* = 121) (data publicly accessible via http://www.cbioportal.org)

Targeted analysis of melanoma mutation data, publicly available online through The Cancer Genome Atlas database (TCGA, http://www.cbioportal.org) was also performed on 278 melanoma samples provided by the TCGA (*n*_T_ = 278) and 121 samples made available through the Broad Institute (Cambridge, MA) (*n*_B_ = 121) [22, 23]. We found that the frequency of mutations in genes among our top 40 most frequently mutated was, overall, comparable to that found in 97.1 % (270 of 278) of TCGA melanoma samples (Fig. 9) and 95 % (115 of 121) of samples provided by the Broad Institute (Fig. 10), although eight of our top 40 (20 %) genes were not available through this database. Notably, however, the prevalence of *BRAF* mutations was higher in the TCGA and Broad Institute cohorts (51 and 63 %, respectively) than in our dataset (42 %). In addition, *MECOM* mutations were found less frequently in the TCGA and Broad Institute cohorts (20 and 16 %, respectively).

**Fig. 11:**
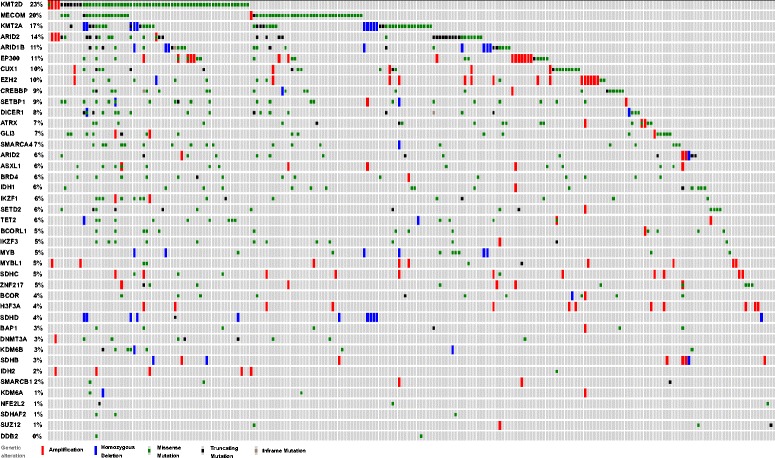
Mutational spectrum among epigenetic genes tested in “Oncopanel” within TCGA cutaneous melanomas (*n* = 278) (data publicly accessible via http://www.cbioportal.org)

**Fig. 12:**
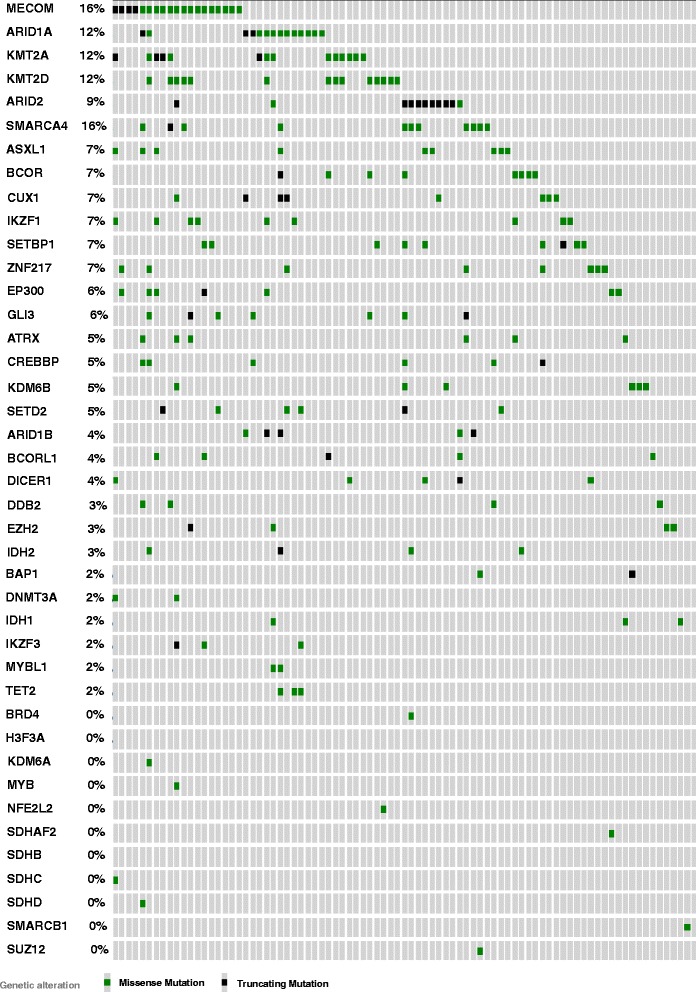
Distribution of mutations in epigenetic genes tested in “Oncopanel” within Broad Institute cutaneous melanomas (*n* = 121) (data publicly accessible via http://www.cbioportal.org)

Furthermore, a focused analysis of mutations in the 41 epigenetic genes tested by our Oncopanel in the TCGA and Broad cohorts revealed a comparable frequency of mutations (Figs. 11 and 12). In keeping with our data, *MECOM*, *MLL2* (*KMT2D*), *MLL* (*KMT2A*), *ARID2*, and *CUX1* were among the most frequently mutated epigenetic genes in the TCGA and Broad Institute melanoma cohorts. In addition, the vast majority of samples in both the TCGA (83.1 %, 231 of 278) and Broad Institute (69.4 %, 84 of 121) cohorts harbored at least one mutation in one of the tested epigenetic genes.

## Discussion

The incidence of melanoma, unlike many other potentially preventable cancers, is steadily increasing worldwide, with an estimated 76,100 new cases diagnosed in the USA in 2014 alone [1]. While accounting for less than 2 % of all skin cancers, melanoma accounts for the vast majority of skin cancer deaths [1]. Major risk factors for melanoma include those that are genetic and environmental in nature, such as having a personal or family history of melanoma, five or more “atypical” nevi, having numerous (>50) melanocytic nevi, fair-colored skin, as well as either a history of blistering sun burns during childhood/adolescence and/or a history of indoor tanning bed use [24]. Epidemiological evidence increasingly implicates UV radiation in melanoma pathogenesis, as was recently detailed in a meta-analysis that demonstrated an increased risk of melanoma in airline pilots and cabin crew (thought to be related to elevated levels of cosmic and UV radiation exposure) [25]. Approximately 10 % of melanomas occur in a familial setting, and germline mutations to a number of genes, including *CDKN2A* (9p21), *CDK4* (12q14), *BAP1* (3p21), *TERT* promoter (5p15) [26], and most recently *POT1* (7q31) [27], have been demonstrated to predispose individuals to developing cutaneous melanoma in addition to other melanoma subtypes (e.g., *BAP1* and metastatic uveal melanoma) as well as numerous atypical melanocytic nevi (*CDKN2A*, *CDK4*) [28]. Collectively, these observations reinforce that the pathogenesis of melanoma is complex, involving both genetic and non-heritable (e.g., environmental) factors.

The most frequently mutated genes within our cohort resemble those reported in previous studies [20, 29] as well as through data available through the Sanger Institute Catalogue Of Somatic Mutations in Cancer (COSMIC) database (http://www.sanger.ac.uk/) and the NIH/NCI TCGA melanoma mutation database (http://www.cbioportal.org) [22, 23, 30]. The well-known oncogenes *NRAS* and *BRAF* as well as the tumor suppressor genes *CDKN2A* and *TP53* (17p13) were among the most frequently mutated genes within our cohort, in keeping with prior studies and datasets [20, 31]. The latter two genes were also among the genes within our cohort containing the highest number and percentage of nonsense mutations, consistent with loss of their tumor suppressive function. While mutations to the *TP53* gene have previously been considered a rare event in melanomagenesis [32], our findings corroborate data from others [20] demonstrating that they may be more common than previously believed. While these large, publically available datasets have the advantage of pooling larger number of patient melanoma samples, at present, they do not allow for the specific characterization of UV-signature mutations, nor do they allow for the direct comparison of such patterns or others between epigenetic and non-epigenetic genes, as our study has done. In addition, these databases do not allow for potential relationships between specific mutations and key melanoma histologic parameters to be explored.

Our dataset and novel analytic approach, with an emphasis on epigenetic mutations, reveal several interesting observations. Firstly, we identified a high frequency of mutations in genes encoding epigenetic regulators in both primary and metastatic cutaneous melanoma patient samples with a statistically significant predilection for mutations bearing the signature of UVB damage. Genes encoding histone-modifying proteins (e.g., *MECOM*, *MLL2*, *SETD2*, etc.), subunits of chromatin-remodeling complexes (e.g., *ARID1B*, *ARID2*), as well as units of the active DNA demethylation pathway (*TET2*, *IDH1*) were the most frequently mutated among this list. We took particular interest in several novel standouts among our list of most commonly mutated epigenetic genes. *MECOM* (3q26), for instance, was among the most frequently mutated genes (3.1 %, 23 of 740 mutations; 36.8 %, 14 of 38 patient samples) within our cohort of patient melanoma samples and was higher than estimated by existing datasets (Figs. 9 and 10) [30].

*MECOM* (MDS1 and EV1 complex locus) encodes ecotropic viral integration site 1 (EVI1), an oncogenic zinc finger transcription factor known to be overexpressed in acute and chronic myeloid leukemia and to correlate with poor patient survival [33, 34]. Interactome analysis has revealed that the EVI1 oncoprotein exerts dynamic nuclear functions and is involved in a number of vital processes, including, but not limited to, transcription regulation, DNA repair, recombination, and mitosis [35]. In addition, EVI1 has been shown to interact with multiple components of the epigenetic machinery, including DNA methyltransferases, histone modifying enzymes, and chromatin-remodeling complexes, including the SWItch/Sucrose NonFermentable (SWI/SNF) nucleosome remodeler [36]. Moreover, gene expression analyses demonstrated a stem cell phenotype in EVI1-overexpressing acute myelocytic leukemia cells, suggesting that this oncoprotein could augment cancer stem cell self-renewal capacity and facilitate disease progression and the development of therapeutic resistance [36]. Similar lines of evidence also suggest that EVI1 may be involved in facilitating chemoresistance in human myeloid leukemias by inducing the CDKN1A/p21/WAF complex [37]. Taken together, we hypothesize that MECOM/EVI1 regulates the epigenetic machinery enabling stem cell-like properties in specific melanoma subpopulations. Additional studies are indicated to explore and evaluate these possibilities.

*MLL2* (or *KMT2D*, 12q13) was the second most frequently mutated epigenetic gene within our cohort. *MLL2* is a member of the myeloid/lymphoid or mixed-lineage leukemia (*MLL*) family genes and encodes a specific histone 3 lysine 4 (H3K4) methyltransferase, which provides an evolutionarily conserved epigenetic mark for active gene transcription [38]. Remarkably, all 16 of 16 mutations in *MLL2* bore the signature of UVB-induced DNA damage. *MLL2* was recently identified to extensively regulate the expression of a number of critical cell signaling pathways, including the p53 pathway and cAMP-mediated signaling, as well as the expression of the retinoic acid-responsive gene *ASB2* [39]. Moreover, and of particular interest to the biology of melanocytes and melanoma, *MLL2* was recently found to associate with the promoters and thereby regulate the expression of S100 alpha (S100A) genes (1q21), which are known to control cell cycle progression and differentiation within the melanocyte [39]. *MLL2* has been frequently implicated in the pathogenesis of a number of human cancers [40–42], and our findings corroborate with recent data [31] suggesting the same may be true for malignant melanoma.

The *IDH1* gene was also among the most commonly mutated epigenetic genes within our collection and was more frequently mutated than reported in prior studies [20]. *IDH1* (2q33) encodes a soluble, cytoplasmic isocitrate dehydrogenase 1 (IDH1) enzyme, an enzyme that converts isocitrate to α-ketoglutarate (also known as 2-oxoglutarate) [43]. Recent evidence suggests significant epigenetic consequences resulting from mutations in this particular enzyme. Loss of IDH1 function results in reduced production of α-ketoglutarate, which is a necessary co-substrate for two critical families of epigenetic regulators, including histone demethylase enzymes and the ten-eleven translocation (TET) family of 5-methylcytosine hydroxylases, otherwise known as Fe (II)-dependent dioxygenase enzymes [44]. Furthermore, mutant IDH1 has been demonstrated to lead to the accumulation of oncometabolite 2-hydroxyglutarate (2-HG), which has been shown to directly inhibit TET function [44, 45]. The TET family enzymes, composed of TET1, TET2, and TET3, are central to the active DNA demethylation pathway [46, 47]. TET2 catalyzes the critical, iterative oxidation steps on the methyl group of 5-methylcytosine to produce 5-hydroxymethylcytosine, 5-carboxylcytosine, and 5-formylcytosine, and ultimately cytosine, after removal of the functional group by thymine DNA glycosylase and the base excision repair pathway [48]. This very recently uncovered pathway has putative significance for maintaining DNA methylation and epigenetic fidelity, which has earned TET the epithet “guardian of the epigenome” [12, 49].

5-hydroxymethylcytosine, an intermediate in DNA demethylation and a product of TET2 function is kinetically the most abundant intermediate in this pathway [50]. Loss of its nuclear immunopositivity has been demonstrated to be a nearly universal hallmark of malignancy [51]. A number of studies have demonstrated that, in most human organ systems, “loss of 5-hydroxymethylcytosine” can distinguish malignant cellular proliferations from the benign, wherein 5-hydroxymethylcytosine expression is retained, with high sensitivity and specificity [13, 51–53]. Such observations bear testament to the pathobiologic significance of TET enzyme dysfunction. Indeed, TET has recently been found to interact in concert with an array of epigenetic regulators, including transcription factors, histone modifying enzymes, chromatin-remodeling complexes, and miRNAs, suggesting a central regulatory role over the epigenome [54]. A recent study documented exceedingly rare somatic *TET2* gene mutations in melanoma [55]. In contrast, six of 38 (15.8 %) of our human melanoma samples had a documented mutation in *TET2*. Moreover, when including *IDH1*-mutant samples, 21 % (8 of 38) of patient samples had at least one mutation affecting the critical DNA demethylation pathway in which these two enzymes participate. That mutation to the gene encoding the TET2 enzyme is only one of many mechanisms that can cause its dysfunction [54] raises the possibility that additional alterations not detectable by genetic sequencing may, too, inhibit the function of this critical regulator of the epigenome and contribute to melanoma virulence.

Secondly, our dataset and analysis also identified a high frequency of UVB-signature mutations among epigenetic genes. The genes encoding the central epigenetic regulator *MECOM/EVI1* (73.9 %, 17 of 23) and H3K4 methyltranferase *MLL2* (100 %, 16 of 16) were found to harbor the greatest overall number of UVB-signature mutations. Both within and outside of the familial/hereditary melanoma setting, ultraviolet light radiation (UVR) is thought to play a major role in the pathogenesis of melanoma. While it is known that most of the mutational burden in melanoma is attributable to the mutagenic effects of UVR [56, 57], our findings raise the possibility that UVR preferentially induces mutations in genes encoding epigenetic regulators and/or that these elements may be involved in mediating a physiologic response. This hypothesis is in keeping with the putative role of epigenetic mechanisms at large in facilitating changes in gene expression in response to environmental cues [12, 58, 59] and could co-exist with pathways known to be involved in the physiologic response of melanocytes to UVR, such as the p53-proopiomelanocortin (POMC) pathway [60]. Interestingly, we found that the well-known oncogenes *BRAF* and *NRAS* had comparatively low frequencies (26.3 and 0 %, respectively) of UVB-signature mutations, a finding that is in keeping with previous studies [20]. In contrast, 81.8 % of *CDKN2A* mutations, 84.6 % of *TP53* mutations, and 83.3 % of *IDH1* mutations resembled the UVB-signature mutation genotype in our cohort.

Genomic evidence that epigenetic regulators may contribute to melanoma pathogenesis was recently highlighted by Hodis et al. [20], who found a high frequency of somatic mutations in chromatin-modifying proteins and other epigenetic regulators as well as a high frequency of UVB-signature mutations in *IDH1* and chromatin-modifying enzymes *ARID2* (a component of the SWI/SNF chromatin-remodeling complex) and *EZH2* (the histone lysine methylase component of Polycomb-group gene silencing complex). Ding et al. [31] also found a high frequency of truncation mutations to chromatin-remodeling genes (*ASXL3*, *MLL2*, *ARID2*) in their cohort of metastatic melanoma cases [31]. Our findings, in addition to data obtained from the COSMIC and TCGA melanoma database (Figs. 11 and 12), contribute additional evidence to this growing body of literature that dysregulated epigenetic complexes and pathways may be more involved in the pathobiology of melanoma than previously recognized. Our preliminary dataset indicate that genomic mutations to epigenetic regulators may be more common than previously appreciated. Altogether, we found that approximately one in five mutations occurred in a gene encoding an epigenetic regulator, with mutations to histone-modifying enzymes being the most common. Moreover, the overwhelming majority (92.1 %, 35 of 38) of our patient samples harbored at least one mutation in an epigenetic regulatory gene with well over half of all patients samples (65.7 %, 25 of 38) having more than one such gene mutated.

While our study provides novel insight into potential epigenetic mechanisms involved in the pathogenesis of melanoma, there are several limitations. Firstly, our modestly sized cohort may under- or overestimate the frequency of certain genetic mutations and also limits our ability to identify a mutational signature, epigenetic or not, that is typical of primary or metastatic melanoma tumors or those that correlate with key histologic parameters that confer a defined level of metastatic potential (i.e., depth, mitotic rate). Further expansion and integration of inter-institutional patient melanoma mutational profiles alongside detailed, case-by-case clinical and histopathologic annotation will be necessary to obtain the resolution necessary to identify these critical relationships. Furthermore, the lack of paired samples within our cohort reduces our ability to identify or directly infer specific genetic mutations that potentially drive metastasis or disease progression. Given that the Oncopanel program at our institution is offered to every oncology patient but is programmatically limited to one test per patient due to economic constraints, our patient samples are unable to be paired. In addition, the panel of epigenetic genes tested in our cohort is not comprehensive, in view of the ever-expanding family of epigenomic regulators [61], and additional sequencing platforms to test for these novel epigenetic genes should be prepared for further investigations. It must also be acknowledged that sequencing techniques do not directly detect chromosomal aberrations, which are known to occur and be involved in the pathogenesis of melanoma [62] and distinguish benign melanocytic lesions from malignant melanoma [63]. Nonetheless, our study highlights the high prevalence of mutations in epigenetic regulators in patient melanoma samples and their tendency to bear the signature of UVB damage. Moreover, our focus on utilizing genetic information to understand dysregulated epigenetic mechanisms in melanoma provides a novel analytical lens that deserves further consideration. Finally, our study demonstrates the clinical application of next-generation sequencing to identify novel mutations in melanoma and may shed light on new, personalized pathogenic mechanisms and unveil potential targets of therapeutic interest [21]. Because epigenetic defects, unlike genetic mutations, are potentially reversible, this area of investigation has tremendous potential for translational and therapeutic application.

Most existing non-immune based targeted therapies attempt to inhibit proteins/enzymes that function predominantly within and beneath the cell membrane and cytoplasm, most of which participate in cell signaling pathways [64, 65]. Most such pathways are poised to influence the expression of select genes, a process that is primarily regulated by transcription factors and, very likely, other functional binding partners, such as chromatin-remodeling enzymes. As we continue to advance our understanding of how epigenetic mechanisms interface with cell signaling pathways and how their dysregulation, independently or combined, contributes to diseases such as cancer, opportunities for therapeutic targeting of pathobiologic epigenetic mechanisms will follow. In parallel, evidence is accumulating that multimodal combination therapy will be critical for the successful treatment of biologically complex malignancies such as melanoma [66]. Targeting epigenetic mechanisms may provide one such adjunctive avenue of attack, emphasizing the importance of delineating the relationships discussed above. Indeed, DNA methyltransferase inhibitors and histone deacetylase inhibitors are the only current examples of existing FDA-approved therapies that function in this manner, although they are not currently in use for melanoma. Our data highlight several candidate epigenetic regulators that deserve further investigation and pathobiological characterization in melanoma. Within our cohort, *MECOM*/EVI1, *MLL2*, and *TET2/IDH* are examples of nuclear epigenetic regulators that we suspect may be involved in the pathogenesis of melanoma and could be involved in enabling stem-like characteristics in select subpopulations. This epigenetic machinery deserves thorough exploration in the context of melanoma pathobiology, and further studies to establish their mutational status in benign nevi are now indicated.

## Conclusions

Herein, we describe the prevalence of somatic mutations present in the genes encoding a spectrum of epigenetic regulators within a cohort of treatment-naïve patient melanoma specimens. We provide direct genomic evidence that epigenetic regulators, including histone/chromatin-modifying enzymes and DNA demethylation enzymes/pathways may be involved in the development and/or progression of melanoma. Moreover, our analysis of patient melanoma samples revealed a high prevalence of mutations in epigenetic regulators with a quantifiable predilection for those associated with UVB damage. In addition, we have identified that *MECOM*, a novel, central epigenetic regulatory gene, and *TET2/IDH1*, critical regulators of DNA demethylation, are frequently mutated in patient melanoma samples.

## Availability of supporting data

The datasets supporting the results of this article are included within the article and its additional file.

## Methods

### Ethics statement

The study was approved by the Institutional Review Board of the Brigham and Women’s Hospital and Dana–Farber Cancer Institute. Patients referred to our institution for the evaluation and management of melanoma were offered an opportunity to participate in this study. Informed consent was obtained from 38 patients to collect a single sample of their primary or metastatic tumor and have it sequenced through the Oncopanel program. Oncopanel was designed and implemented to provide an opportunity for every oncology patient seen and evaluated at the institution to have their tumor tested. However, because its use is not currently being reimbursed, it is programmatically limited to only one test per patient at the present time. All 38 patients had not previously received any form of chemotherapy or radiation for the treatment of their melanoma.

DNA was isolated from formalin-fixed melanoma tissue using standard methods. Samples were incubated in proteinase K overnight, followed by subsequent purification of the DNA (QIAamp DNA Mini Kit, QIAGEN, Gaithersburg, MD). DNA concentration was assessed using PicoGreen dsDNA detection (Life Technologies, Carlsbad, CA). Targeted NGS was performed using a cancer genomic assay to detect mutations in the exonic regions of 275 cancer genes previously implicated in tumorigenesis and 91 intronic regions across 30 of the 275 genes (Oncopanel, BWH/DFCI, Additional file 1). The complete coding sequence of the target genes was captured using a solution-phase Agilent SureSelect hybrid capture kit (AgilentTechnologies, Inc., Santa Clara, CA), and massively parallel sequencing was performed on an Illumina HiSeq 2500 sequencer (Illumina, Inc. San Diego, CA). Mutation calls were performed using Mutect and GATK software (Broad Institute, Cambridge, MA).

Data analysis was performed using an internally developed bioinformatics Pipeline (Riker, REF) that was composed of reconfigured publically available tools (GATK, MuTect, Indelocator, Oncotator) and internally developed algorithms (VisCap Cancer [REF], Phaser, BreaKmer3). Reads obtained from pooled samples were demultiplexed using Picard (http://picard.sourceforge.net/command-line-overview.html), aligned to the Human Genome Reference Consortium reference sequence GRCh37p13 (BWA5), and duplicate reads were subsequently removed (Picard). GATK6 was used to refine the alignments near insertion/deletion (indel) sites. Single nucleotide variants (SNVs) were called using MuTect7, and indels were called using Indelocator (http://www.broadinstitute.org/cancer/cga/indelocator). Annotation was performed using Oncotator. Because tumor tissues were sequenced without a paired normal from the corresponding patients, additional informatic steps were taken to identify and account for common single nucleotide polymorphisms (SNPS): any SNP present at >0.1 % in Exome Variant Server (NHLBI GO Exome Sequencing Project [ESP], Seattle, WA; URL: http://evs.gs.washington.edu/EVS/) or present in dbSNP was filtered. However, variants also present in the COSMIC mutation database were rescued for manual review. Samples with a mean target coverage of <50X were failed and excluded from further analysis. Individual variants present at <10 % allele fraction or in regions with <50X coverage were flagged for manual review and evaluated/interpreted by the reviewing laboratory scientists and molecular pathologists based on a variety of factors, including, but not limited to, overall tumor percentage, read depth, complexity of alteration, and evidence for associated copy number alterations.

Mutation data was queried with Microsoft Sequel and quantitatively analyzed with Microsoft Excel. Individual gene mutation frequencies were calculated based on the total number of mutations as well as based on the total number of patients. Genes encoding proteins involved in DNA methylation/demethylation, histone modification, chromatin remodeling, or processing of non-coding RNAs (such as microRNAs) were categorized as “epigenetic regulatory genes”. Gene functions were determined by referencing the genetic database (accessible at: http://ghr.nlm.nih.gov/) provided by the United States National Library of Medicine and the National Institute of Health and supplemented by identifying recently published literature available on PubMed documenting specific epigenetic function by a particular gene or its expressed protein. Quantitative data was analyzed using MedCalc version 13.2 (MedCalc Software, Ostend, Belgium) and StatPlus version 5.8.2 (AnalystSoft Inc.). Statistical methods were primarily descriptive and based on proportions and percentages. The proportion of epigenetic genes is presented with a 95 % exact binomial confidence interval. Proportions of epigenetic genes or mutations present within particular cohorts were compared using chi-square analysis. All *p* values were two-sided, with a *p* < 0.05 considered statistically significant.

Additional mutation data was obtained from the publically available databases (http://www.cbioportal.org, Memorial Sloan Kettering Cancer Center, New York, NY) provided by the National Institute of Health (NIH) and National Cancer Institute (NCI) for the purposes of comparison [22, 23]. In addition, to better understand the relationships between mutated epigenetic regulators, we performed Ingenuity Pathway Analysis (Qiagen, Redwood City, CA) to visualize direct relationships between specific epigenetic regulators.

## Additional file

Additional file 1:
**List of the 275 cancer gene exons as well as the 91 introns from 30 of these genes sequenced by our “Oncopanel” cancer genomic assay.**

## Abbreviations

α-KG: alpha-ketoglutarate
5-mC: 5-methylcytosine
5-hmC: 5-hydroxymethylcytosine
5-caC: 5-carboxylcytosine
*ARID1B*: AT rich interactive domain 1B (SWI1-like)
*ARID2*: AT rich interactive domain 2 (RFX-like)
*ASB2*: ankyrin repeat and SOCS box protein 2
*ASXL3*: additional sex combs like 3
*BAP1*: BRCA1 associated protein-1 (ubiquitin carboxy-terminal hydrolase)
*BRAF*: B-Raf proto-oncogene, serine/threonine kinase
BWH: Brigham and Women’s Hospital
C: cytosine
*CDK4*: cyclin-dependent kinase 4
*CDKN2A*: cyclin-dependent kinase inhibitor 2A
*CREBBP*: CREB binding protein
COSMIC: Catalogue of Somatic Mutations in Cancer
*CUX1*: cut-like homeobox 1
DFCI: Dana-Faber Cancer Institute DNA, Deoxyribonucleic acid
DNMT: DNA methyltransferase
*EZH2*: enhancer of zeste homolog 2
FDA: United States Food and Drug Administration
G: guanine
HDAC: histone deacetylase
IDH: gene encoding isocitrate dehydrogenase
*KMTD*: lysine (K)-specific methyltransferase 2D
*KMT2A*: lysine (K)-specific methyltransferase 2A
*MECOM*: MDS1 and EV1 complex locus
*MLL(2)*: mixed-lineage leukemia protein (2) miRNA, MicroRNA
NCI: National Cancer Institute
*NF1*: neurofibromin 1
NGS: next-generation sequencing
NIH: National Institute of Health
*NRAS*: neuroblastoma RAS viral (v-ras) oncogene homolog
POMC: p53-proopiomelanocortin
*POT1*: protection of telomeres 1
*SETD2*: SET domain containing 2
SWI/SNF: SWItch/Sucrose NonFermentable
T: thymine
TCGA: The Cancer Genome Atlas
*TERT*: telomerase reverse transcriptase
TET: ten-eleven translocase
*TP53*: tumor protein p53
UVR: ultraviolet radiation
