# Supplementary material for: Targeted next-generation sequencing reveals high frequency of mutations in epigenetic regulators across treatment-naïve patient melanomas
Source: Clin Epigenetics. 2015 Jun 9;7(1):59. doi: 10.1186/s13148-015-0091-3 (PMC4517542; doi:10.1186/s13148-015-0091-3)
Supplement: Additional file 1: — List of the 275 cancer gene exons as well as the 91 introns from 30 of these genes sequenced by our “Oncopanel” cancer genomic assay. [file 13148_2015_91_MOESM1_ESM.docx]

**ADDITIONAL FILE**

**Additional file 1.** List of the 275 cancer gene exons sequenced in our cancer genomic assay as well as the 91 introns from 30 of these genes. Genes encoding epigenetic regulators have been bolded.

*ABL1, AKT1, AKT2, AKT3, ALK, ALOX12B, APC, AR, ARAF,* ***ARID1A, ARID1B, ARID2, ASXL1,*** *ATM,* ***ATRX****, AURKA, AURKB, AXL, B2M,* ***BAP1****, BCL2, BCL2L1, BCL2L12, BCL6,* ***BCOR, BCORL1****, BLM, BMPR1A, BRAF, BRCA1, BRCA2,* ***BRD4****, BRIP1, BUB1B, CARD11, CBL, CBLB, CCND1, CCND2, CCND3, CCNE1, CD274, CD58, CD79B, CDC73, CDH1, CDK1, CDK2, CDK4, CDK5, CDK6, CDK9, CDKN1A, CDKN1B, CDKN1C, CDKN2A, CDKN2B, CDKN2C, CEBPA, CHEK2, CIITA,* ***CREBBP****, CRKL, CRLF2, CRTC1, CRTC2, CTNNB1,* ***CUX1****, CYLD,* ***DDB2****, DDR2,* ***DICER1****, DIS3, DMD,* ***DNMT3A****, EGFR,* ***EP300****, EPHA3, EPHA5, EPHA7, ERBB2, ERBB3, ERBB4, ERCC2, ERCC3, ERCC4, ERCC5, ESR1, ETV1, ETV4, ETV5, ETV6, EWSR1, EXT1, EXT2,* ***EZH2****, FAM46C, FANCA, FANCC, FANCD2, FANCE, FANCF, FANCG, FAS, FBXW7, FGFR1, FGFR2, FGFR3, FGFR4, FH, FKBP9, FLCN, FLT1, FLT3, FLT4, GATA3, GATA4, GATA6, GLI1, GLI2,* ***GLI3****, GNA11, GNAQ, GNAS, GPC3, GSTM5,* ***H3F3A****, HNF1A, HRAS, ID3,* ***IDH1, IDH2****, IGF1R,* ***IKZF1****,* ***IKZF3****, JAK2, JAK3,* ***KDM6A****,* ***KDM6B****, KDR, KIT, KRAS, LMO1, LMO2, LMO3, MAP2K1, MAP2K4, MAP3K1, MAPK1, MCL1, MDM2, MDM4,* ***MECOM****, MEF2B, MEN1, MET, MITF, MLH1,* ***MLL, MLL2,*** *MPL, MSH2, MSH6, MTOR, MUTYH,* ***MYB, MYBL1,*** *MYC, MYCL1, MYCN, MYD88, NBN, NF1, NF2,* ***NFE2L2****, NFKBIA, NFKBIZ, NKX2-1, NOTCH1, NOTCH2, NPM1, NRAS, NTRK1, NTRK2, NTRK3, PALB2, PARK2, PAX5, PDCD1LG2, PDGFRA, PDGFRB, PHF6, PHOX2B, PIK3C2B, PIK3CA, PIK3R1, PIM1, PMS1, PMS2, PNRC1, PRAME, PRDM1, PRF1, PRKAR1A, PRKCI, PRKCZ, PRKDC, PRPF40B, PRPF8, PSMD13, PTCH1, PTEN, PTK2, PTPN11, RAD21, RAF1, RARA, RB1, RBL2, REL, RET, RFWD2, RHPN2, ROS1, RPL26, RUNX1, SBDS,* ***SDHAF2, SDHB, SDHC, SDHD, SETBP1, SETD2,*** *SF1, SF3B1, SH2B3, SMAD2, SMAD4,* ***SMARCA4, SMARCB1****, SMC1A, SMC3, SMO, SOCS1, SOX2, SOX9, SRC, SRSF2, STAG1, STAG2, STAT3, STAT6, STK11, SUFU,* ***SUZ12****, SYK, TCF3, TCF7L1, TCF7L2, TERT,* ***TET2****, TNFAIP3, TP53, TSC1, TSC2, U2AF1, VHL, WRN, WT1, XPA, XPC, XPO1,* ***ZNF217****, ZNF708, ZRSR2.*

Intronic regions of: ABL1, AKT3, ALK, BCL2, BCL6, BRAF, CIITA, EGFR, ETV1, EWSR1, FGFR1, FGFR3, FUS, IGH@, IGK@, IGL@, JAK2, MLL, MYC, NPM1, PAX5, PDGFRA, PDGFRB, RAF1, RARA, RET, ROS1, TRA@, TRB@, TRG@.
